# A quantum dot fluorescent microsphere based immunochromatographic strip for detection of brucellosis

**DOI:** 10.1186/s12917-021-02760-w

**Published:** 2021-01-23

**Authors:** Yufang Kong, Huiyu Wang, Shaoqiang Wu, Jizhou Lv, Lin Mei, Huifang Zhou, Xiangmei Lin, Xueqing Han

**Affiliations:** 1 Institute of Animal Inspection and Quarantine, Chinese Academy of Inspection and Quarantine, 100176 Beijing, China; 2 People’s Hospital of Jiaxiang, Jiaxiang County, Jining City, 272400 Shandong Province China

**Keywords:** Brucellosis, Quantum dots fluorescent microspheres, Immunochromatographic strip test

## Abstract

**Background:**

Brucellosis is a serious zoonosis disease that frequently causes significant economic loss in animal husbandry and threatens human health. Therefore, we established a rapid, accurate, simple and sensitive fluorescent immunochromatographic strip test (ICST) based on quantum dots (QDs) for detection the antibodies of *Brucella* infection animals serum.

**Results:**

The test strips were successfully prepared by quantum dot fluorescent microspheres (QDFM) as tracers, which were covalently coupled to an outer membrane protein of *Brucella* OMP22. The outer membrane protein OMP28 and monoclonal antibodies of OMP22 were separately dispensed onto a nitrocellulose membrane as test and quality control lines, respectively. The critical threshold for determining negative or positive through the ratio of the fluorescent signal of the test line and the control line (H_T_ / H_C_) is 0.0492. The repeatability was excellent with an overall average CV of 8.78%. Under optimum conditions, the limit of detection was 1.05 ng/mL (1:512 dilution). With regard to the detection of brucellosis in 150 clinical samples, the total coincidence rate of ICST and Rose Bengal plate test (RBPT) was 97.3%, the coincidence rate of positive samples was 98.8%, the coincidence rate of negative samples was 95.3%, the sensitivity of RBPT is 1:32, and no cross reaction with the sera of other related diseases was observed.

**Conclusion:**

In our present study, the QDFM has promising application for on-site screening of brucellosis owing to its high detection speed, high sensitivity, high specificity and low cost.

## Background

Brucellosis is a highly infectious zoonosis and poses serious threats to human health [1]. *Brucella* can infect humans in many ways, for example, contact with infected livestock and wildlife, and consumption of meat products and milk products infected with *Brucella* or incidental exposure to live attenuated vaccine and so on [2–5]. *Brucella* contributes to abortions, infertility, placenta retention, still birth or weak offspring, and poor reproductive performance of animals, which results in huge economic losses for livestock farmers [6]. Currently, there is no effective method to prevent this disease, therefore, early diagnosis and monitoring are very essential.

Traditional detection methods of *Brucella* are pathogen isolation identification, serological diagnosis and molecular biology, which have a few defects [7]. The pathogen isolation identification method produces qualitative and quantitative results, but the method requires strict laboratory conditions and poses potential exposure risk to performers. The serological diagnostic methods including the Rose Bengal plate test (RBPT), standard tube agglutination test (SAT) and enzyme-linked immunosorbent assay (ELISA) need the whole cell or whole smooth lipopolysaccharides (S-LPS) as the antigen. Moreover, these methods may cause false positives and cross-reactivity with other Gram-negative bacteria [8–10]. Molecular biology methods such as polymerase chain reaction (PCR) [11], real time PCR (qPCR) [12], provide qualitative and quantitative results with good accuracy and sensitivity. However, these methods require expensive instruments and professional operators. They are time consuming and easy to produce aerosol pollution [13]. Therefore, it is extremely important to establish a rapid, accurate and sensitive method to detect brucellosis [7].

In previous studies, the colloidal gold test strip method showed low sensitivity and species limitation. Dmitriy et al established *Brucella* colloidal gold antibody test strip can only detect bovine serum and the serum dilution limit of detection was only 1:250 [14]. Recently, a new labelled and more sensitive method was developed with fluorescent microspheres [15, 16]. Therefore, in this study, we developed a diagnostic for brucellosis by immunochromatography labeled with QDFM.

## Results

### Optimization of the coating concentration for the NC membrane

**Fig. 1:**
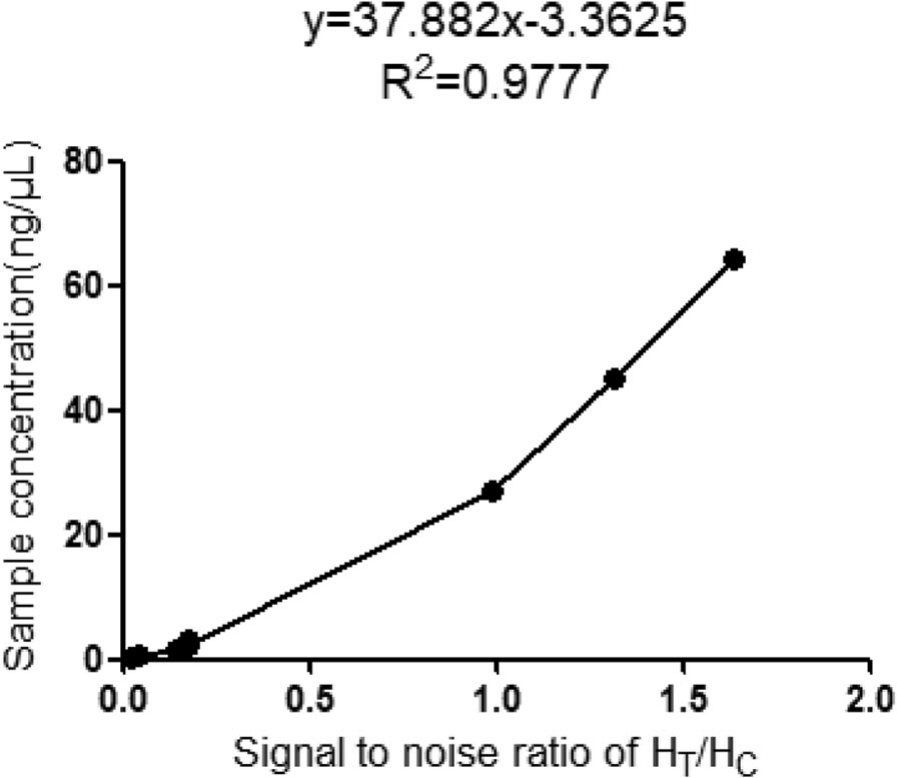
Optimization of the coating concentration for the NC membrane

The optimal coating concentration of the test line and control line were selected as 1 mg/mL and 0.5 mg/L, respectively. As shown in Fig. 1, there was good correlation between H_T_/H_C_ (x) and the sample concentration (y). The linear regression equation was y = 37.882x - 3.3625 and the correlation coefficient was 0.9777, indicating the feasibility of ICST for detecting brucellosis.

### Limit of detection

**Fig. 2:**
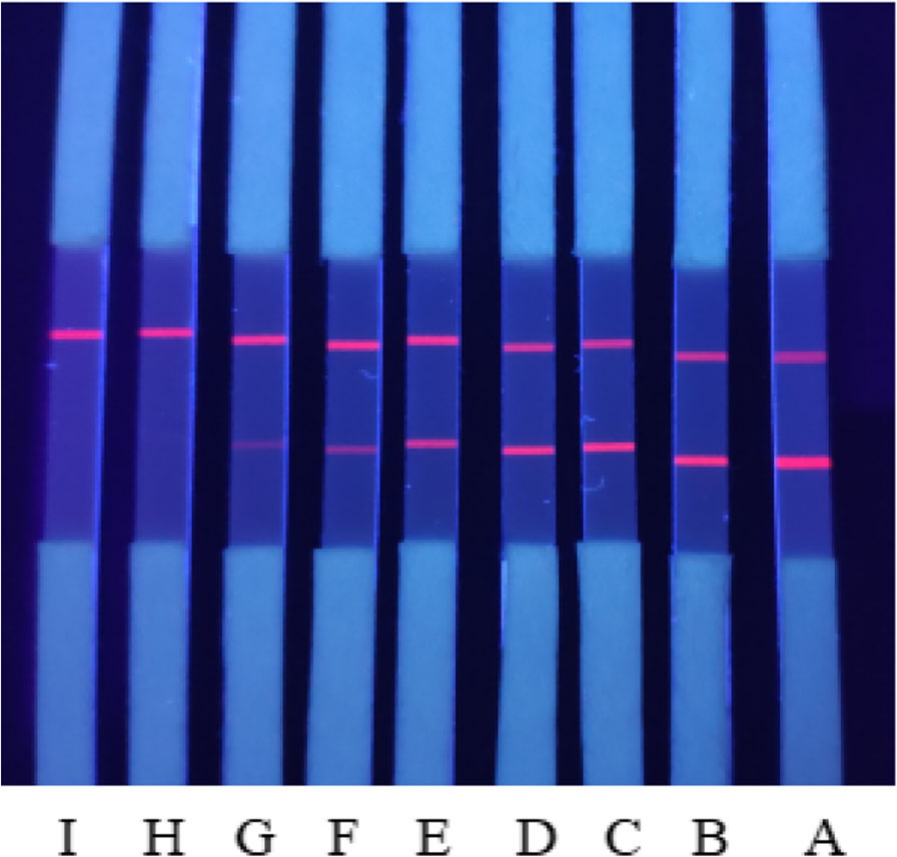
The extreme detection limit of ICST for *Brucella* standard positive serum

The standard curve was established with serial 2-fold dilutions of *Brucella* positive serum from 1:4 to 1:1024, which were detected by. As shown in Fig. 2, the results can be read with naked eyes using a UV lamp and the fluorescent intensity of the test line gradually decreased. For low-antibody samples, the fluorescence intensity of the test line was weaker than the control line. At a dilution of 1:512, the fluorescence intensity was weakened, and the limit of detection was 1.05 ng/mL.The fluorescent line disappears at a dilution of 1:1024. As shown in Table 1, these values can also be accurately detected by a fluorescence reader.

**Table 1:** Sensitivity assay of ICST testing of brucellosis

Dilution	H_T_	H_C_	H_T_/ H_C_
1∶4	65,376	12,324	5.3048
1∶8	56,124	13,632	4.1171
1∶16	55,735	18,346	3.0380
1∶32	47,775	20,434	2.3380
1∶64	34,152	33,784	1.0109
1∶128	26,789	31,892	0.8400
1∶256	16,995	31,145	0.5457
1∶512	8667	31,972	0.2711
1∶1024	56	32,754	0.0017

### Threshold and specificity test

To determine the threshold, 50 healthy serum samples were tested by the ICST and their results suggested that the ICST threshold is 0.0492. The H_T_/H_C_ value ≥0.0492 (Table 2) indicated a positive assay of the ICST. The brucellosis samples displayed H_T_/H_C_ values greater than the threshold, indicating the positive results. The *Y.enterocolitica* O:9*, E.coli* O:157, *Salmonella Dublin* samples all displayed H_T_/H_C_ values less than the threshold, indicating the negative results and there was no cross reaction with the sera of other diseases (Table 3).

**Table 2:** The threshold assay of ICST with 50 healthy *Brucella* negative serum samples from bovine and sheep

Samples	H_T_/H_C_				
Negative serum samples	0.0154	0.0004	0.0156	0.0151	0.0145
	0.0123	0.0269	0.0287	0.0269	0.0238
	0.0208	0.0212	0.0260	0.0332	0.0127
	0.0143	0.0037	0.0101	0.0414	0.0011
	0.0187	0.0116	0.0070	0.0303	0.0123
	0.0362	0.0312	0.0109	0.0116	0.0103
	0.0244	0.0128	0.0125	0.0117	0.0201
	0.0316	0.0146	0.0281	0.0219	0.0018
	0.0151	0.0267	0.00148	0.0327	0.0333
	0.0007	0.0139	0.0044	0.0312	0.0031
	Mean = 0.0177				
	Standard deviation =0.0105				
	Threshold = 0.0492

**Table 3:** Specificity of the ICST for brucellosis

Samples	H_T_	H_C_	H_T_/H_C_	Result
Brucellosis	54,654	12,350	4.4254	(+)
*Y.enterocolitica* O:9	1196	32,035	0.0373	(−)
	1023	31,989	0.0320	(−)
*E.coli* O:157	1325	30,425	0.0435	(−)
	1146	32,147	0.0356	(−)
	1158	32,201	0.0360	(−)
	1123	31,987	0.0351	(−)
*Salmonella Dublin*	1427	31,043	0.0460	(−)
	1277	32,176	0.0400	(−)

### Detection of brucellosis in clinical samples using ICST and RBPT

The results of 150 samples were detected by the Fluorescence Reader. The coincidence rate was calculated by comparing with RBPT results. As shown in Table 4, the total coincidence rate of ICST and RBPT was 97.3% [(85 + 61)/150]. Compared with RBPT, the positive coincidence rate of ICST was 98.8% [85/(85 + 1)] and the negative coincidence rate of ICST was 95.3% [61/(61 + 3)]. As shown in Table 5, the sensitivity of RBPT is 1:32.

**Table 4:** Clinical sample detection with ICST and RBPT

ICTS	RBPT		Total
	Positive	Negative	
Positive	85	3	88
Negative	1	61	62
Total	86	64	150

**Table 5:** Sensitivity assay of RBPT testing of brucellosis

Dilution	Result
1∶2	(+)
1∶4	(+)
1∶8	(+)
1∶16	(+)
1∶32	(+)
1∶64	(−)
1∶128	(−)
1∶256	(−)

### Repeatability assay of the ICST for brucellosis

The results showed that the ICST maximum CV value of all samples was 8.78%, and the average value was 6.16%. All the CV values of the above results are less than 10%, indicating that the diagnostic procedures for brucellosis based on QDFM detection technology was repeatable (Table 6).

**Table 6:** Repeatability of the ICST for brucellosis

C (ng/mL)	H_T_/H_C_				
	Repeat1	Repeat2	Repeat3	Mean	CV(%)
0	0.0171	0.0175	0.0177	0.0174	1.42
1	0.0478	0.0455	0.0484	0.0472	2.65
5	0.0641	0.0626	0.0697	0.0654	4.60
10	0.1684	0.1661	0.1449	0.1598	6.62
15	0.6964	0.6316	0.6802	0.6694	4.11
20	1.1364	1.1056	1.0006	1.0809	5.38
25	1.9127	1.5876	1.7925	1.7643	7.61
30	1.9263	1.7422	1.6239	1.7641	7.05
35	2.0472	1.8752	2.3045	2.0756	8.50
40	2.3329	2.1006	2.1923	2.2086	4.33
45	2.9876	2.5969	2.4273	2.6706	8.78
50	3.0015	3.0314	3.1425	3.0585	1.98

## Discussion

At present, it has been more than 100 years to develop effective methods for diagnosis of brucellosis, but brucellosis is still a recurring disease and is prevalent again in many parts around the world [17]. *Brucella* infections are easily transmitted to humans, causing acute febrile illness -- undulant fever -- which may progress to a more chronic form and can also produce serious complications affecting the musculo-skeletal, cardiovascular and central nervous systems [OIE Terrestrial Manual chapter 3.01.04]. The most rational approach for preventing human brucellosis is the control and elimination of the infection in animals [https://www.who.int/zoonoses/diseases/brucellosis/en/]. Therefore, it is important to select the main diagnostic antigen of brucellosis and establish a rapid diagnostic method to prevent and treat the disease.

Serology immunological detection technology mainly relies on LPS antigen, however, related studies have shown that LPS antigen has the high cross-reactivity of with several Gram-negative bacteria. Therefore, the LPS antigen is not appropriate for specificity diagnosis of brucellosis [18–20]. In recent years, many researchers are looking for a better diagnostic antigen such as outer membrane proteins (OMPs) to replace LPS to improve the specificity of immunological detection technology [21–24]. OMPs are exposed on the bacterial surface, which may be closely related to the virulence of brucellosis [25]. Lindler et al identified a group of non-LPS immunogens as OMPs, which can be used for vaccine development and brucellosis diagnosis [26]. One of these OMPs, OMP22, has many advantages, such as being highly conserved among all *Brucella* species and almost identical in amino acid sequence to OMP25. In a previous clinical study, the absence of OMP25 or OMP22 proteins was demonstrated to lead to a striking decrease in the virulence of B. *ovis* PA in mice [27]. Another OMPs, OMP28, is a conserved protein that presents in at least four *Brucella* species including *B. melitensis*, *B. abortus*, *B. suis*, and *B. ovis*. The protein has been fully studied and can be used as a vaccine candidate or as an antigen for serological diagnosis [28–30]. The studies showed that the rOMP28-based I-ELISA had high sensitivity and specificity in the diagnosis of brucellosis in bovine sera [31–34]. Lim et al established an ELISA method for detecting bovine brucellosis antibodies by coating rOMP28 antigen. The sensitivity, specificity and accuracy of the method are 96.7, 95.4 and 96.2%, respectively [28]. In our present study, we also used the OMP22 and OMP28 as the diagnostic antigen to test brucellosis antibodies by immunochromatography.

*Brucella* OMPs are generally expressed in the form of inclusion bodies in *E. coli*, and the refolding rate of inclusion bodies is low, which cannot meet the requirements of this test. Therefore, the pCold-TF DNA vector containing a 48kD fusion tag was used in this study to express OMP22 and OMP28 in the supernatant in *E. coli*. Considering that the large fusion tag will affect the immunogenicity of the protein, the fusion tag was removed by HRV 3C Protease. And then the target protein was purified by combining with His-Tag containing medium.

Recently, many researchers have focused on the development of quantum dot fluorescent microspheres (QDFM) immunochromatography, which has been widely used in the field of biological and chemical detection. Compared with other detection technologies, these immunoassay methods have many advantages such as fast detection speed, good efficiency, strong specificity, high sensitivity and simple operation [35]. For example, Taranova et al established a QD-based immunochromatographic analysis method for the detection of several antibiotics in milk [36]. This labelled technology forms hundreds or even thousands of particles by encapsulating or connecting to other materials to form nanoparticles. It has the unique characteristics of good light stability and biocompatibility, long fluorescence lifetime, wide excitation spectrum, narrow emission spectrum and adjustable size. With these advantages, QDFM is expected to become an applied immunolabeling technology [37, 38]. Compared with other labeling technologies, QDFM amplify the light signal of antigen-antibody specific binding and improve the sensitivity [39, 40]. The method has the advantages of short detection time, no need for sophisticated instruments, simple operation and low cost [41, 42].

QDFM test strips have strict requirements for the immunogenicity of labelled proteins. Dan et al developed a method to detect *Brucella* by combining QD and magnetic beads with different polyclonal antibodies. The method requires 105 min and the limit of detection was 10^3^ CFU/mL [43]. The established method in this study takes only 10–15 min to obtain the test results and its limit of detection is 1.05 ng/mL. After the coating concentration of the NC membrane was optimized, H_T_/H_C_ (x) and sample concentration (y) showed a good correlation (Fig. 2).

As is shown in Table 3, QDMF can be used for *Brucella* antibodies detection in real samples with high specificity. The test results of *Brucella* serum samples were positive, while the test results of *Yersinia enteritidis* O:9, *E. coli* O:157 and *Salmonella Dublin* were all negative, indicating that the method has good specificity. As Table 4 shows, ICST shows high feasibility in the 150 clinical serum samples assay and the total coincidence rate of ICST and RBPT was 97.3%. Compared with RBPT, the positive coincidence rate of ICST was 98.8% and the negative coincidence rate of ICST was 95.3%. This further shows that the detection method can obtain ideal results in the detection of *Brucella* clinical samples.

## Conclusion

In this study, we presented QDFM tagged OMP22 to facilitate detection of *Brucella* antibodies in standard and clinical samples of only a few microliters using ICST. The limit of detection was 1.05 ng/mL (1:512), the total coincidence rate of ICST and RBPT was 97.3%, the positive coincidence rate was 98.8%, the negative coincidence rate was 95.3%, the repeatability was good and the overall average CV value is 8.78%, the sensitivity of RBPT is 1:32 and no cross reaction with the sera of other related diseases was observed. However, the quantitative research of this method needs to be further studied.

## Methods

### Materials and reagents

Quantum dot fluorescent microspheres were purchased from Invitrogen Corp (Carlsbad, CA, USA). A Rose Bengal plate test (RBPT) was obtained from the China Institute of Veterinary Drug Control. Bovine serum albumin (BSA), tween-20, polyvinyl pyrrolidone (PVP), sodium azide, tris base (TB), 2-(4-Morpholino) ethanesulfonic acid (MES), 1-(3-dimethylaminopropyl)-3-ethylcarbodiimide hydrochloride (EDC) and N-hydroxysuccinimide (NHS) were purchased from Sigma Chemical Co. (St. Louis, MO, USA). The test strip materials, including nitrocellulose (NC) membranes (Millipore Hiflow-95) and glass cellulose membranes (Product number 8951), were purchased from Shanghai Jiening Biotechnology Co., Ltd. (Shanghai, China).

### Apparatus

The BioDot XYZ dispensing platform (BioDot, Richmond, CA, USA) was used to dispense reagents to nitrocellulose membrane, conjugate pad and an automatic cutter (BioDot, Richmond, CA, USA) was used to cut the strips. A fluorescent strip reader JN615 was purchased from Shanghai Jie Ning Biological Co., Ltd. (Shanghai, China). A 365-nm hand held UV lamp (American Precision Co., Ltd., USA) was used to test strips.

### Samples and biological materials

Bovine *Brucella* negative and positive standard sera were purchased from the China Institute of Veterinary Drug Control. Positive sera of *Y.enterocolitica* O:9(2 goat serum)*, E.coli* O:157(4 bovine serum), *Salmonella Dublin* (2 sheep serum) and 50 healthy negative bovine and sheep (30 bovine serum, 20 sheep serum) were preserved in the Chinese Academy of Inspection and Quarantine. A total of 150 clinical serum samples (68 bovine serum, 44 sheep and 38 goat serum) were kindly provided by the Animal Husbandry Bureau of Ningxia Hui Autonomous Region (Table 7). *Brucella* OMP22 and OMP28 and monoclonal antibodies of OMP22 were prepared by our laboratory.

**Table 7:** Clinical positive serum sample background

ID	Animal species	species
A1-A39	bovine	*B. abortus*
B1-B23	goat	*B. melitensis*
C1-C26	sheep	*B.ovis*
D1-D29	bovine	/
E1-E15	goat	/
F1-F18	sheep	/

### Preparation of QDFM conjugates protein

The protein is coupled to QDFM through carboxyl activation. Transfer the commercial QDFM solution (100 μL) into a centrifuge tube and prepare OMP22-QDFM using EDC and NHS as cross-linking agents. The mixture solution was dissolved in MES buffer to produce a final concentration of 0.5 mg/mL EDC and 0.2 mg/mL NHS. The solution was vortexed for 30 min and then reacted at 37°Cfor 15 min. Then, 100 μL of OMP22 (0.1 mg/mL) was added and the mixture was reacted for 2–4 h under slow stirring at room temperature. Fifty μL of 10% BSA was added and the mixture was incubated at room temperature for 30 min. The resulting QDFM conjugate was washed 3 times by centrifugation at 8000 g for 20 min. The QDFM-OMP22 conjugate was resuspended in 1 mL of 20 mM Trise solution (TB, pH 8.5) containing 0.5% BSA, 2% sucrose, 0.2% Tween-20, Triton 405-X and kept at 4 °C until use [44].

### Assembly of the QDFM test strip

**Fig. 3:**
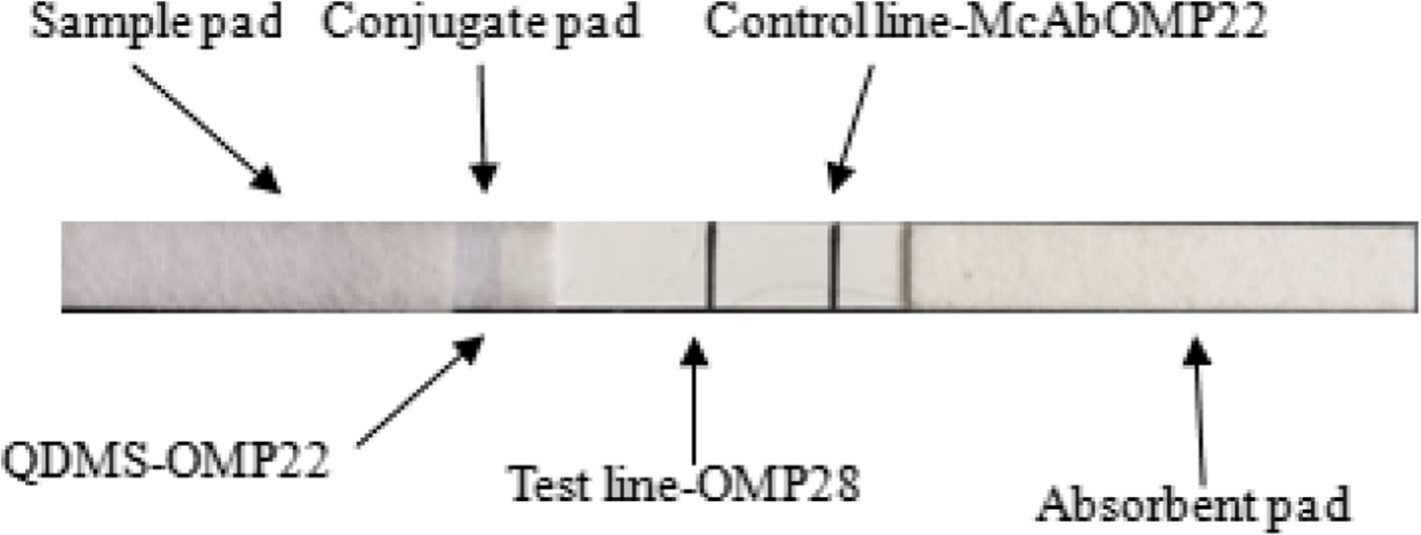
Schematic diagram of the QDFM based multiplex ICST. The control line was coated with McAb against OMP22 and the test lines was coated with OMP28. The conjugate pad was QDFM functional OMP22

The test strip is composed of four parts: sample pad, conjugate pad, nitrocellulose membrane and absorbent pad. The sample pad is soaked in 20 mM TB (pH 8.5) buffer containing 5% sucrose, 0.5% BSA, 0.01% PVP-40, 2% Tween-20 and 0.02% NaN3. And then dried at 70 °C for 2 h and stored at room temperature. Paste the test strip components on the PVC backplane in turn and overlap the two components by 2-mm to ensure that the test sample solution can migrate to the entire assembled test strip. In our present study, QDFM labeled *Brucella* OMP22 was dispensed onto the conjugate pad and then the pad was dried at 37 °C overnight and stored at 4 °C. 0.03 mL of OMP28 (1.5, 1, 0.75 mg/mL) and 0.03 mL of McAb OMP22 (1, 0.75, 0.5 mg/mL) were dispensed onto the nitrocellulose membrane as test and control lines, respectively, and the strip was dried at 37 °C for 2 h. Finally, the whole assembled strip was cut into a 5-mm width and 80-mm length using a BIO-DOT strip cutting machine (Fig. 3). Eight brucellosis positive serum samples with different concentrations were tested to determine the NC membrane coating concentrations. The corresponding concentrations of the samples were 0.169 ng/μL, 0.666 ng/μL, 1.35 ng/μL, 2.11 ng/μL, 3.06 ng/μL, 27.06 ng/μL, 45.2 ng/μL, 64.2 ng/μL, respectively.

According to the three detection antibody analysis protocols described by Sotnikov et al our research plan is similar to the author’s second protocol. The extramembrane protein OMP22 binds to the antibody in the serum sample and is captured by OMP28 on the detection line to form an OMP22-Ab-OMP28 immune complex [45].

### Sensitivity, threshold, specificity, feasibility and repeatability test

The standard cure was established with serial 2-fold dilutions of *Brucella* positive serum from 1:4 to 1:1024 and ICST was used determine the limit of detection.

Fifty healthy bovine and sheep serum samples were tested as negative controls to determine the threshold of results. The 365 nm handheld UV lamp is used to initially observe the results of the test strip, and then use a fluorescence reader to record the ratio of the test line signal to the control line signal (H_T_/H_C_). Calculate the H_T_/H_C_ threshold of ICST by the following formula: Threshold = average ± 3 × standard deviation.

ICST was used to detect *Yersinia enteritidis* O: 9, *E. coli* O: 157 and *Salmonella Dublin* positive serum samples and the results were recorded with a fluorescence reader. According to the size of the threshold to determine the specificity of the test strips.

In order to evaluate the feasibility of the test strip for detecting brucellosis antibodies, 150 clinical serum samples of brucellosis were collected from the Animal Husbandry Bureau of Ningxia Hui Autonomous Region. All samples were pretreated with 0.01 M Tris-HCl (pH 9.5) buffer containing 0.9% NaCl and 0.05% Tween-20 for 15 min. One hundred fifty clinical samples were tested using ICST and the coefficients of the test results were compared with commercial RBPT. Serial 2-fold dilution of bovine *Brucella* positive standard sera from 1:2 to 1:256 were used in order to determine the sensitivity of RBPT [46].

The repeatability of ICST was tested by 11 serially diluted standard brucellosis positive serum samples concentrations ranging from 50 ng/mL to 1 ng/mL and 1 negative serum. Each sample was detected for three times to calculated coefficient of variation (CV). The CV was calculated by dividing the mean of three measurements by the standard deviation to determine the repeatability.

## Data Availability

The data sets used and/or analyzed during the current study are available from the corresponding author on reasonable request.

## Abbreviations

ICST: Immunochromatographic strip test
RBPT: Rose Bengal plate test
QDFM: Quantum dot fluorescent microspheres
SAT: Standard tube agglutination test
ELISA: Enzyme-linked immunosorbent assay
S-LPS: Smooth lipopolysaccharides
PCR: Polymerase chain reaction
qPCR: Real time PCR
OMPs: Outer membrane proteins
TB: Tris base
NC: Nitrocellulose
MES: 2-(4-Morpholino) ethanesulfonic acid
EDC: 1-(3-dimethylaminopropyl)-3-ethylcarbodiimide hydrochloride
NHS: N-hydroxysuccinimide
BSA: Bovine serum albumin
CV: Coefficient of variation
PVP: Polyvinyl pyrrolidone
